# Germline mutations of the *INK4a-ARF* gene in patients with suspected genetic predisposition to melanoma

**DOI:** 10.1038/sj.bjc.6601503

**Published:** 2004-01-20

**Authors:** N Soufir, J J Lacapere, G Bertrand, E Matichard, R Meziani, D Mirebeau, V Descamps, B Gérard, A Archimbaud, L Ollivaud, F Bouscarat, M Baccard, G Lanternier, P Saïag, C Lebbé, N Basset-Seguin, B Crickx, H Cave, B Grandchamp

**Affiliations:** 1 Laboratoire de Biochimie Hormonale et Génétique, Hôpital Bichat-Claude Bernard, 46 rue henri Huchard, Paris 75018, France; 2 Inserm U 410 Faculté Bichat-Claude Bernard, Paris 75018, France; 3 Laboratoire de Biochimie-Génétique, Hôpital Robert Debré, Paris 75018, France; 4 Dermatology Department, Hôpital Bichat-Claude Bernard, Paris 75018, France; 5 Dermatology Department, Hôpital Saint-Louis, Paris 75010, France; 6 Dermatology Department, Hôpital Percy, Clamart 92140, France; 7 Dermatology Department, Hôpital Ambroise Paré, 92 Boulogne Billancourt, France

**Keywords:** *INK4a*-ARF, Cdk4, melanoma genetics, germline mutation, deletion

## Abstract

Germline anomalies of the *INK4a-ARF* and *Cdk4* genes were sought in a series of 89 patients suspected of having a genetic predisposition to melanoma. Patients were selected based on the following criteria: (a) familial melanoma (23 cases), (b) multiple primary melanoma (MPM; 18 cases), (c) melanoma and additional unrelated cancers (13 cases), (d) age at diagnosis less than 25 years (21 cases), and (e) nonphoto-induced melanoma (NPIM; 14 cases). Mutations of *INK4a-ARF* and *Cdk4* were characterised by automated sequencing, and germline deletions of *INK4a-ARF* were also examined by real-time quantitative PCR. Seven germline changes of *INK4a-ARF*, five of which were novel, were found in seven patients (8%). Four were very likely to be pathogenic mutations and were found in three high-risk melanoma families and in a patient who had a pancreatic carcinoma in addition to melanoma. Three variants of uncertain significance were detected in one MPM patient, one patient <25 years, and one NPIM patient. No germline deletion of *INK4a-ARF* was found in 71 patients, and no *Cdk4* mutation was observed in the 89 patients. This study confirms that *INK4a-ARF* mutations are infrequent outside stringent familial criteria, and that germline *INK4a-ARF* deletions are rarely involved in genetic predisposition to melanoma.

Familial melanomas comprise from 8 to 12% of all cutaneous malignant melanoma cases (Greene, 1999). Two highly penetrant melanoma-predisposing genes have been identified to date, *INK4a-ARF* and *Cdk4* (Hussussian *et al*, 1994; Kamb *et al*, 1994; Zuo *et al*, 1996).

The *INK4a-ARF* gene on chromosome 9p21 encodes two structurally distinct tumour-suppressor proteins by virtue of different 5′ exons spliced in different reading frames to common exons 2 and 3. Exons 1*α*, 2, and 3 encode p16^INK4*a*^, while exon 1*β*, spliced to exons 2 and 3 in a different reading frame and transcribed using a different promoter, encodes p14^ARF^ protein (ARF, also called p19^ARF^ in mice). P16^INK4*a*^ is part of the G1–S cell cycle checkpoint mechanism that involves the retinoblastoma-susceptibility tumour suppressor protein (pRB). PRB protein, in its unphosphorylated state, inhibits the progression of the cell cycle from the G1 to the S phase by sequestering the transcription factor E2F1. Phosphorylation of pRB by the cyclin-dependent-kinases CDK4 and 6 (CDK4-6/D kinases) releases E2F1 and allows progression through the G1–S checkpoint. P16^INK4*a*^ is a specific inhibitor of CDK4 and 6, and thus, inactivation of p16^INK4*a*^ allows cells to escape cell cycle arrest in G1.

The other product of the *INK4a-ARF* locus, p14^ARF^, also acts as a tumour suppressor (Sherr, 2000). Mice lacking ARF, but with intact p16^INK4*a*^, develop tumours (Kamijo *et al*, 1997), while transfection of ARF into some carcinoma cell lines results in marked growth inhibition (Simon *et al*, 1999; Yang *et al*, 2000). ARF mediates G1 and G2 arrest at least partly by its interaction with MDM2, a protein that binds to both TP53 and pRB. MDM2 targets TP53 for degradation by ubiquitination (Ashcroft and Vousden, 1999) and also inhibits pRB growth-regulatory function. ARF binds to MDM2 and promotes its degradation (Pomerantz *et al*, 1998; Zhuang *et al*, 1998), resulting in stabilisation and accumulation of TP53 protein and also of its downstream target p21, an inhibitor not only of CDK4 and 6 but also of other CDKs.

Multiple studies have shown that germline mutations in the *INK4a-ARF* gene are found on average in approximately 25% of melanoma-prone families (reviewed in (Hayward, 2000; Piepkorn, 2000). The frequency of *INK4a-ARF* mutations in melanoma probands increases with (i) the number of affected relatives, (ii) the presence of multiple melanomas in the same patient, (Soufir *et al*, 1998a; Holland *et al*, 1999) and (iii) a history of pancreatic cancer cases in the family (Goldstein *et al*, 1995).

In contrast, mutations of *Cdk4* appear to be a rare cause of inherited susceptibility to melanoma. Four germline alterations have been described in *Cdk4* to date, in four melanoma-prone kindreds (Zuo *et al*, 1996; Soufir *et al*, 1998a; Holland *et al*, 1999) and in one melanoma patient with no known family history (Guldberg *et al*, 1997). Two mutations occur in exon 2 of *cdk4* at the same codon (Arg24Cys, Arg24His) and functional analysis of the Arg24Cys mutant protein reveals that it is deficient in binding to p16^INK4*a*^, but is capable of binding cyclin D and phosphorylating pRB.

Whereas germline mutations in the *INK4a-ARF* gene are uncommon in unselected melanoma patients from the general population (Aitken *et al*, 1999), the prevalence of *INK4a-ARF* mutations in patients suspected of having a genetic predisposition to melanoma outside a familial context remains to be clarified.

Therefore, in this work, we hypothesise that in addition to patients with familial melanoma, some patients could have an inherited predisposition to melanoma and might harbour germline mutations of *INK4a-ARF*. These patients include those who had multiple primary melanomas (MPM), melanoma associated with another cancer, melanoma developing at a young age, or nonphoto-induced melanomas (NPIM).

This hypothesis is strengthened by the fact that germline mutations of the *INK4a-ARF* gene have been detected in some MPMs (MacKie *et al*, 1998; Monzon *et al*, 1998; Hashemi *et al*, 2000; Auroy *et al*, 2001), and could also predispose to other types of cancers, such as pancreatic cancer (Borg *et al*, 2000), epidermoid carcinoma (Yarbrough *et al*, 1996), breast cancer (Borg *et al*, 2000), or multiple myeloma (Dilworth *et al*, 2000) in melanoma families.

The proposition that patients who develop melanoma at a young age harbour a strong predisposition to melanocyte neoplasia is consistent with Knudson's hypothesis that cancers arising at a very young age may result from mutations to key regulatory genes passed through the germline model for the incidence of retinoblastoma. Finally, melanomas considered to be nonphoto-induced (NPIM) include (i) melanomas located on nonphoto-exposed sites (sun-protected skin areas or mucous localisations) and (ii) subungual and acral lentiginous melanomas that are considered to be particular subtypes of melanoma, because, in contrast to other subtypes, ultraviolet irradiation is not a major factor in their development (Kato *et al*, 1996; Saida, 2001), thus suggesting genetic factors in their onset.

Therefore, the specific aim of this study was to assess the prevalence of *INK4a-ARF* and *Cdk4* mutations among different subgroups of patients with a possible hereditary predisposition to melanoma. Here, we report that *INK4a-ARF* mutations are predominantly found in high-risk melanoma kindreds, confirming previous reports. However, we also found that *INK4a-ARF* mutations are rarely present in individuals with suspected genetic predisposition to melanoma (MPM, melanoma arising before the age of 25 years, NPIM) without a family history of melanoma.

## PATIENTS AND METHODS

### Selection of patients

The present study was performed from 1999 to September 2002. Patients were enrolled at the Saint Louis (50%), Bichat-Claude Bernard (45%), Ambroise Paré (5%), and Percy Hospitals (5%), which are located in or near Paris city. In all, 89 patients were prospectively enrolled in the study, of which 10% were incident cases. The inclusion criteria were: (1) familial melanoma (FAM; 23 cases) defined as the presence of at least two melanoma cases in first- or second-degree relatives (all cases confirmed by pathological reports); (2) multiple primary melanomas (MPM) defined as the presence of at least two primary melanomas in the same patient confirmed by pathological reports (18 cases); (3) melanoma in young patients defined as melanoma in patients younger than 25 years (21 cases); (4) nonphoto-induced melanoma (14 cases) defined by either melanomas located on nonphoto-exposed sites (sun-protected skin areas or mucous localisations), and/or subungual and acral lentiginous melanomas, which are considered to be particular subtypes of melanoma; and (5) melanoma associated with another cancer (13 cases). For familial melanoma cases, only the proband was enrolled.

Written informed consent allowing peripheral blood sampling and genetic analysis was obtained for each patient enrolled in the study. Adoption and xeroderma pigmentosum cases were excluded.

For each patient included, clinical information was obtained by a dermatologist and from medical records: family history of melanoma, age at diagnosis of melanoma, tumour location, histopathological classification and Breslow thickness, diagnosis of multiple primary melanomas or other cancer, nevus count, and the presence of clinically atypical mole syndrome (AMS), defined as at least 50 nevi >2 mm in diameter and including at least three atypical nevi.

### DNA PCR and sequencing

Genomic DNA was extracted from peripheral blood lymphocytes as described by Miller *et al* (1988). The *INK4a-ARF* locus comprises four exons, coding for two alternative transcripts: p16^INK4*a*^ (exons 1*α*, 2 and 3) and p14^ARF^ (exons 1*β*, 2 and 3). PCR was performed on 50–100 ng of extracted DNA using the following cycling conditions: 35 cycles at 94°C (30 s), Tm at 62°C (30 s) and 72°C (1 min), and 10% DMSO (dimethylsulphoxide). The primer sequences for exons 1*α*, 1*β*, and 2 are listed in Table 1

**Table 1:** PCR primers used for automated sequencing of *INKA-ARF* coding exons (1*α*, 1*β*, 2, 3) and *Cdk4* exon 2

**Primer localisation**	**Primer name**	**Sequences (5′–3′)**
*Sequencing primers*		
Exon 1*α* p16^INK4A^	P16F1	GCT CGG CGG CTG CGG AGA GG
	P16R1	TCC AGA GTC GCC CGC CAT CCC
Exon 2 INK4a-ARF	P16F2	GGG TCT GCT TGG CGG TGA GG
	P16R2	CGG GCT GAA CTT TCT GTG CTG GA
Exon 1*β* p14^ARF^	P14F1	TGC GTG GGT CCC AGT CTG CAG
	P14R1	ACC GCG GTG GAG GCT TCC CAT
Exon 2 Cdk4	CDK4F1	GGA TGG GAT GCT GGT GGT GTT
	CDK4R1	TTC CCT TTA CTC CCC ACG CCC

. Coding and flanking intron sequences of exon 2 of the *Cdk4* gene, which include residues previously shown to be mutated in familial melanoma (Zuo *et al*, 1996; Soufir *et al*, 1998b) were amplified using the primer pair Cdk4F1 and Cdk4R1 (Table 1).

The PCR products were purified with the PCR product presequencing kit according to the manufacturer's protocol. All fragments were sequenced with the Applied Biosystems (ABI) BigDye Terminator Cycle Sequencing Ready Reaction Kit according to the manufacturer's instructions, and sequencing reactions were analysed on an ABI 3100 automated sequencer.

### *INK4a-ARF* deletion analysis by real-time quantitative PCR

INK4*a*-ARF deletion analysis was performed as recently described for the characterisation of mono- and biallelic 9p21 deletions in childhood acute lymphoblastic leukaemia (Bertin *et al*, 2003). Real-time quantitation was performed using the SYBR Green I dye as a fluorescent signal. The dye binds specifically to the minor groove of double-stranded DNA, allowing the detection of PCR product formation (Ginzinger, 2002).

Two targets were amplified on 9p21: *p16*^*INK4a*^ exon 3 and *p14*^*ARF*^ exon 1*β*. One single-copy sequence was used as a reference sequence: 8q11 SST (Single Sequence Tag), mapping at 8q11. A volume of 5 *μ*l of DNA was added to the PCR reaction mixture containing 1 × SYBR Green buffer (Applied Biosystems), 300 nM forward and reverse primers, 5 mM MgCl_2_ (3 mM for 8q11 SST), 200 *μ*M dNTP, and 0.6 U of AmpliTaq Gold (Applied Biosystems) in a final volume of 25 *μ*l.

Each series of PCR reactions included two negative controls containing water in place of DNA, one control containing 15 ng of HeLa DNA, and a five-point standard curve. The standard curve was established using serial dilutions of normal PBMC in Tris (10 mM)-EDTA (1 mM) buffer, ranging from 10 to 0.02 ng *μ*l^−1^ (corresponding to 50 ng to 0.1 ng of DNA analysed per well). The same dilutions were used for all targets and reference sequences. PCR was performed on the ABI PRISM 7700 Sequence detector system (Applied Biosystems). All analyses were performed in duplicate. The PCR amplification profile was as follows: initial denaturation at 95°C for 10 min, followed by 40 cycles of denaturation at 95°C for 10 s, and combined annealing and extension at 65°C for 1 min. Detection of the fluorescent product was carried out at the end of the extension period. To confirm amplification specificity, the PCR products from each primer pair were subjected to a melting-curve analysis and subsequent agarose gel electrophoresis. The concentration of each gene was calculated based on the respective calibration curve. The relative copy numbers of *p16*^*INK4a*^ and *p14*^*ARF*^ were then obtained by calculating the ratio of the result obtained for each target to the 8q11 SST value. The normalised ratio of each target on 8q11 SST was expected to be close to 1 if no deletion was present. Childhood acute lymphoblastic leukaemia samples carrying somatic deletion at the *INK4a-ARF* locus were used as positive controls (Bertin *et al*, 2003).

## RESULTS

### Patient characteristics

Patients were categorised into five different melanoma subgroups: (a) familial melanoma (FAM, 23 cases), (b) multiple primary melanoma (MPM, 18 cases), (c) melanoma and additional unrelated cancers (13 cases), (d) age at diagnosis less than 25 years (21 cases), and (e) nonphoto-induced melanoma (NPIM, 14 cases). The numbers of patients in each group, median age at diagnosis, patient's nevus count, and the presence of AMS are listed in Table 2

**Table 2:** Characterisation of the different melanoma subgroups

	**MM subgroup**	**Number of patients**	**Median age at diagnosis**	**Naevus count >50**	**AMS**
1	FAM	23	43 (18−74)	11 (48)	4 (17)
2	MPM	18	42 (19−70)	8 (47)	4 (22)
3	MM <25 years	21	21 (14−25)	11 (50)	6 (28)
4	MM+other cancer	13	54 (27−76)	3 (27)	1 (8)
5	NPIM	14	58 (42−73)	4 (28)	1 (7)

MM = malignant melanoma; FAM = melanoma families; MPM = multiple primary melanoma; NPIM = nonphoto-induced melanoma.

.

Of the 23 melanoma-prone families, two had four melanoma-affected members, five had three affected members, and 16 had two affected members. In all, 17 families had at least two first-degree-related affected members, whereas the six remaining families had second-degree-related affected members. Five families (all with two first-degree-related affected members) comprised at least one member with multiple melanomas, and five families comprised one patient who developed a melanoma before the age of 25 years.

Among the 18 MPM cases selected, 14 developed two melanomas, and four developed three distinct melanomas. The median age for the onset of the first melanoma was 42 years (range 19–70).

In total, 14 patients had a melanoma classically considered not to be photo-induced (NPIM). Of these, three patients had a melanoma localised on the digestive tract (anal and colon, one and two patients, respectively). Four patients had a melanoma localised on the buttocks, and two had a melanoma localised on the genital organs. Four patients had an acrolentiginous melanoma, of which three were subungual, and one was located on the sole of the foot. One patient had a melanoma of the scalp.

Totally, 13 patients developed another cancer in addition to melanoma: seven patients had glandular carcinomas (mammary adenocarcinoma: three cases; thyroid carcinoma, one case; prostate carcinoma, one case; uterus carcinoma, one case; colon carcinoma, one case; pancreatic carcinoma, one case); four other patients developed multiple basal cell carcinomas; one patient had a meningioma.

### Mutational analysis of *INK4a-ARF* and *Cdk4* genes

In the present study, we identified two previously reported mutations in the *INK4a-ARF* gene and five novel *INK4a-ARF* mutations (Table 3

**Table 3:** *INK4a-ARF* germline mutations characterised in melanoma patients

**Patients**	**MM subgroup**	***INK4a-ARF* mutation**	**Effect on p16^INK4a^**	**Effect on p14^ARF^**
1	FAM	Ins 24 bp exon 1*α*	Duplication	—
2	FAM	c**C**c>c**G**c	Pro70**Arg**	Ala84ala
3	FAM	**A**ct>**C**ct	Thr77**Pro**	His**91Pro**
4	MM+K pancreas	g**C**c>g**T**c	Ala57**Val**	Arg71Arg
5	MM <25 years	Ga**C**>ga**T**	Asp105Asp	Arg**120Cys**
6	MPM	**G**cg>**A**cg	Ala5**Thr**	—
7	NPIM	a**C**a>a**T**a	nd	—
		−25 bp atg ex 1*α*		

The sequence is written as 5′ → 3′ for the coding strand; the mutated base is written in uppercase letter. The text in bold indicates nucleotide and amino-acid changes. Abbreviations: Ins = insertion; MM = malignant melanoma; FAM = familial melanoma; MPM = multiple primary melanoma; NPIM = nonphoto-induced melanoma; n.d. = not determined; — = no effect.

).

Four mutations likely to be pathogenic were detected in three melanoma families and in a patient who had a pancreatic cancer in addition to melanoma.

The first mutation, present in a melanoma-prone family comprising three affected members (FAM1), is a 24 bp insertion located at nucleotide 23 of the *p16*^*INK4a*^ mRNA, and is believed to arise due to unequal crossing-over between the two 24 bp repeats naturally present in the wild-type sequence. The result is the addition of eight (duplicated) aminoacids at the flexible N-terminal end of the translated protein outside the ankyrin motifs, and has also been reported to not affect p16^INK4*a*^ activity (Monzon *et al*, 1998). The functional significance of this in-frame insertion is uncertain as it occurs outside of the ankyrin domains. However, it is noteworthy that it segregates with melanoma in four melanoma kindreds (two in Australia, one in the UK, and one in the United States) (Walker *et al*, 1995; Flores *et al*, 1997; Harland *et al*, 1997). In our case, the mutation is present in the unaffected mother of the proband, but unfortunately the other two melanoma-affected members (the grandmother and her mother) could not be tested because they are deceased.

**Figure 1:**
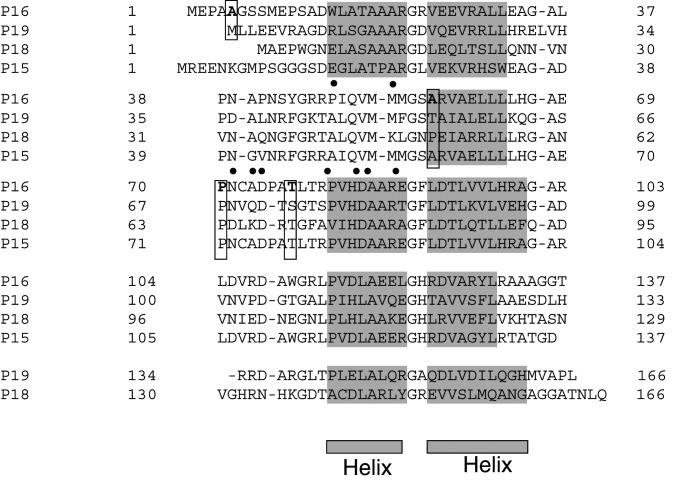
Localisation of the *p16*^*INK4a*^ missense mutations within the p16^INK4a^ protein. The four ankyrin repeats of human p16^INK4a^ are aligned with the corresponding regions of human p18^INK4c^, p19^INK4d^, and p15^INK4b^. C-terminus region of p16^INK4a^, p18^INK4c^, and p19^INK4d^ were cut. Boxes indicate the positions of the *p16*^*INK4a*^ mutations identified in the present study: residue Pro70 is highly conserved, whereas residue Thr77 is semiconserved, and Ala5 and Ala57 residues are not conserved. Filled circles indicate localisation of other *p16*^*INK4a*^ mutations previously shown to induce a loss of function. The SWISS-PROT database was used to delineate the seven alpha helices (shaded in grey).

The second mutation, a C>G substitution, is located in exon 2 of the *INK4a-ARF* gene, and changes the p16^INK4*a*^ reading frame (Pro70Arg), whereas it is neutral for p14^ARF^ (Ala84Ala; Table 3). It was found in a family with two first-degree-related affected members, the proband and her father (FAM2). It should be noted that the proband developed four distinct melanomas at the ages of 38, 45, and 54 years. This mutation is localised in the sixth beta turn of the p16^INK4*a*^ protein, connecting the second and third ankyrin repeats, at a conserved residue (Figure 1

). The replacement of a proline residue by an arginine residue at this position might therefore disturb the spatial arrangement of the two adjacent *α* helixes (Figure 1).

**Figure 2:**
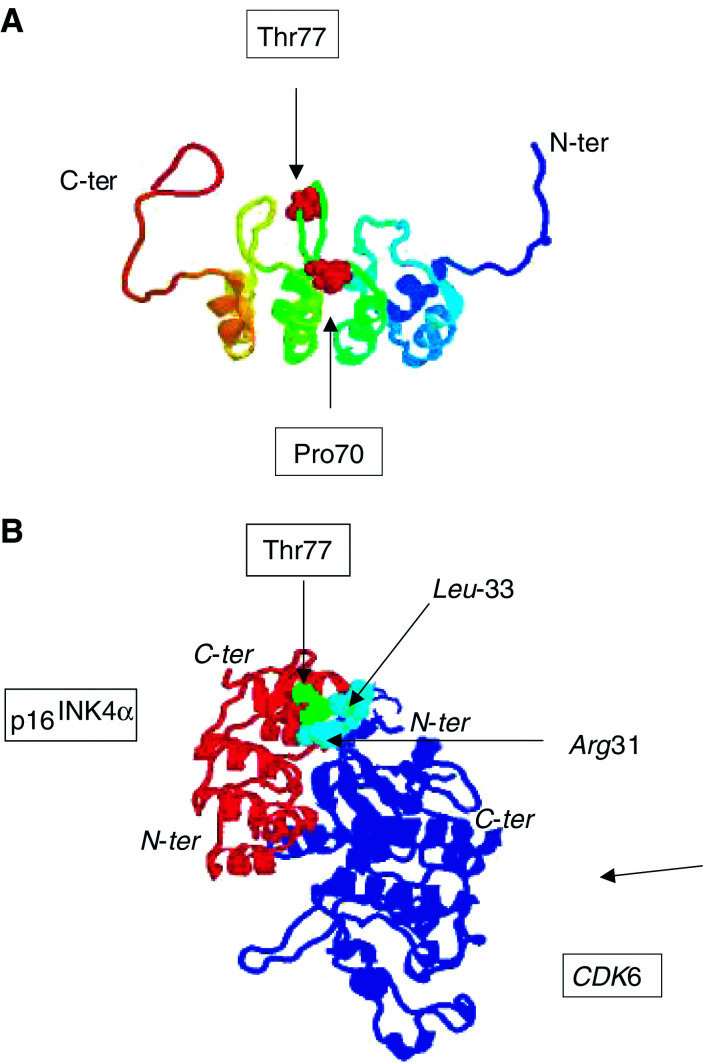
(**A**) Localisation in the overall p16^INK4a^ protein structure (PDB 2A5E) of the two *p16*^*INK4a*^ mutants found in FAM2 and FAM3. (**B**) Localisation of p16^INK4a^ Threonine 77 residue in the p16^INK4a^-CDK6 complex (PDB 1B17) and identification of possible partner residues in CDK6.

The third mutation, an A>C substitution, is also located in *INK4a-ARF* exon 2 and alters both p16^INK4*a*^ (Thr77Pro) and p14^ARF^ (His91Pro) reading frames. It was found in a family with four first-degree-related affected members (FAM3). Binding and recognition between p16^INK4*a*^ and CDK4 or CDK6 proteins are mediated primarily by hydrogen-bond networks (Russo *et al*, 1998), with several of the residues that participate in these interactions being mutated in cancer (Figure 1). Thr77 is localised in the second loop of p16^INK4*a*^, in close contact with Leu33 and Arg31 residues of CDK6 involved in p16^INK4*a*^ binding (Figures 1 and 2b

). Therefore, the introduction of a proline residue at this position is certainly deleterious because of the distortion of the p16^INK4*a*^ tertiary structure as a consequence of a misinteraction with the CDK4 protein. In addition, the missense mutation on p14^ARF^, His91Pro, is located in the nucleolar domain of the protein, therefore being potentially deleterious for p14^ARF^ function.

The fourth mutation was detected in a patient who had a pancreatic carcinoma in addition to cutaneous melanoma, and is characterised by a C>T substitution at nucleotide 170 that changes the p16^INK4*a*^ reading frame (Ala57Val), whereas it is neutral for p14^ARF^ (Arg71Arg; Table 3). This mutation is located in the second ankyrin repeat of p16^INK4*a*^ at the beginning of the *α* helix (Figure 1), in close contact with conserved hydrogen-bond networks that have a central role in CDK6 binding (Russo *et al*, 1998). Yet, although Ala57 is not a conserved amino acid across species, its proximity to the alpha helix of the second ankyrin repeat may place spatial constraints that may be altered with a valine substitution. In addition, it has previously been described as a germline mutation in the case of familial melanoma (Soufir *et al*, 1998b), and as a somatic mutation in the case of acute lymphoblastic leukaemia (Quesnel *et al*, 1995), therefore being certainly deleterious.

Three *INK4a-ARF* changes of uncertain significance were detected in three patients. A C>T substitution was found in a young melanoma patient (a woman aged 24 years). This mutation is also localised in *INK4a-ARF* exon 2, but has no effect on p16^INK4*a*^ (Asp105Asp), whereas it induces a C-terminal amino-acid substitution (Arg120Cys) in the p14^ARF^ protein, at a nonconserved codon between human and mice ARF cDNA. A missense mutation in *p16*^*INK4a*^ exon 1*α* (substitution G>A) was found in a woman who had two distinct melanomas. This novel mutation (Ala5Thr) resides at the N terminus of the p16^INK4*a*^ protein and has not been previously reported as a polymorphism in any *p16*^*INK4a*^ mutational studies. Finally, a *p16*^*INK4a*^ variant was also found in a 64-year-old man with an NPIM (localised on genital organs). This C>T substitution was localised 25 bp upstream from the *p16*^*INK4a*^ initiator translation site and was previously described as a somatic mutation in a skin tumour from a xeroderma pigmentosum patient (Soufir *et al*, 2000), suggesting that it had a pathogenic role.

Three previously described *p16*^*INK4a*^ polymorphisms were confirmed in this study. Ala148Thr was observed in seven patients (8%). This nonsynonymous polymorphism was previously found in 4% of the Utah population (Kamb *et al*, 1994), 3% of CEPH parents (Hussussian *et al*, 1994), in 11% of 131 Australian melanoma kindreds (Holland *et al*, 1999), and was recently excluded as a melanoma/nevus susceptibility allele (Bertram *et al*, 2002).

The G/C transversion at nucleotide 500 in the 3′ untranslated region (UTR) within exon 3 was identified in 23 of the 89 patients (26%). This polymorphism has previously been reported to be present in 11% of CEPH parents (Ueki *et al*, 1994), and found to be associated with familial melanoma risk in Queensland (Aitken *et al*, 1999), but not with sporadic melanomas (Kumar *et al*, 2001). The C/T polymorphism at nucleotide 540 in the 3′ UTR was detected in 14 patients (allelic frequency 8.4%), at a frequency lower than the one previously found in sporadic melanoma (14%) (Kumar *et al*, 2001).

Sequencing of *Cdk4* exon 2 failed to detect mutations in the coding sequence in any patient.

### *INK4a-ARF* deletion analysis by real-time quantitative PCR

Large germline deletions are rare in familial cancers. Nevertheless, a proportion of melanoma families linked to the 9p21 locus do not harbour germline mutations of *INK4a-ARF*, and three large deletions have been reported in hereditary predisposition to melanoma and nervous system tumours, two involving both *p16*^*INK4a*^ and *p14*^*ARF*^ (Bahuau *et al*, 1998), and one restricted to exon 1*β* of *p14*^*ARF*^ (Randerson-Moor *et al*, 2001). Therefore, this suggests that germline lesions of *INK4a-ARF* as a genetic mechanism in predisposition to melanoma may need to be reassessed. We therefore investigate *p16*^*INK4a*^ and *p14*^*ARF*^ deletion status by using real-time quantitative PCR. The normalised ratio of each target (exon 3 of *p16*^*INK4a*^, exon 1*β* of *p14*^*ARF*^) on 8q11 SST was close to 1, indicating that no *INK4a-ARF* germline deletion was present in any of the 71 patients examined.

## DISCUSSION

To investigate more precisely the role of genetic mechanisms in the aetiology of melanoma, we performed a mutational analysis of *INK4a-ARF* and *Cdk4* genes (exon 2) and a deletion analysis of *INK4a-ARF* in a series of 89 cases of melanoma with suspected hereditary predisposition. Seven patients (8%) had a total of seven different germline changes in *INK4a-ARF*, in three melanoma kindreds, and four sporadic melanomas (one melanoma associated with a pancreatic cancer, one melanoma occurring before the age of 25 years, one multiple primary melanoma, and one melanoma localised to nonphoto-exposed skin). No mutation was found in exon 2 of *Cdk4*.

Three *p16*^*INK4a*^ very likely pathogenic mutations were detected in three out of 23 (13%) melanoma families. This mutational frequency is lower than that previously found in French melanoma families, 48% (Soufir *et al*, 1998a). Yet, the inclusion criteria in the former study were much more stringent than in the present one; indeed, melanoma families were selected upon stringent criteria: more than three cases; or two cases in first-degree-related individuals, one of them being below 50 years old, with one additional criterion (multiple primary melanoma in one affected member, pancreatic cancer in the family) (Soufir *et al*, 1998a). It should be noted that in the present study, *INK4a-ARF* mutations were found in families comprising, respectively, four, three, and two cases of melanomas. In the latter family (FAM1), the proband developed multiple primary melanomas. No mutations were found in families with second-degree-related affected members. Therefore, our study further confirms that there is a positive correlation between the frequency of *INK4a-ARF* germline mutation and the strength of family history of melanoma as recently reported (Holland *et al*, 1999).

One *p16*^*INK4a*^ pathogenic mutation was detected in a patient who had a melanoma associated with a pancreatic cancer, but no family history of melanoma (Table 3). These data confirm that the occurrence of both pancreatic cancer and melanoma, in the same patient, signals an inherited susceptibility to cancer, and that this predisposition is, in some cases, due to germline *p16*^*INK4a*^ mutations (Lal *et al*, 2000). However, we found no *INK4a-ARF* mutation in 12 other subjects who had a sporadic melanoma associated with various other cancers. Although our group is too small to draw definitive conclusions, our data are in accordance with a recent report in which no mutation was detected in 27 melanoma patients who also had another cancer (Alao *et al*, 2002).

Three *INK4a-ARF* mutations were also found for which an association with a genetic predisposition to melanoma remains uncertain (Table 3), but that were devoid of 100 ADNs ethnically matched controls previously studied (Soufir *et al*, 1998a). The first one, a novel *p16*^*INK4a*^ missense mutation, was found in one out of 18 (5%) MPM patients. This result could be in agreement with published reports, in which 9.6% (3/31), 11% (2/17), 9% (9/100), and 3% (2/65) of MPM patients were carriers of *INK4a-ARF* germline mutations (MacKie *et al*, 1998; Monzon *et al*, 1998; Hashemi *et al*, 2000; Auroy *et al*, 2001). However, Ala5Thr is most likely a polymorphism as the amino terminus of p16^INK4*a*^ prior to the start of the ankyrin repeats is poorly conserved and is believed to have no effect on the stability of p16^INK4*a*^. Nevertheless, it has recently been shown that some p16^INK4*a*^ mutants failed to induced growth arrest despite retaining normal binding to CDK4 (Becker *et al*, 2001), suggesting that *p16*^*INK4a*^ mutations outside the ankyrins motifs may confer a predisposition to melanoma through a mechanism not yet identified.

The second variant affects only the p14^ARF^ reading frame and was found in one of 21 (5%) young melanoma patients without a family history of melanoma. To date, *p16*^*INK4a*^ germline mutations were characterised in only two out of 55 MM patients less than 30 years, but that both had a history of familial melanoma (Whiteman *et al*, 1997; Tsao *et al*, 2000). Together with our data, this shows that the *INK4a-ARF* gene is rarely involved in genetic predisposition to melanoma in young patients with no familial history of melanoma. However, in the present case, a pathogenic effect of this mutation is suggested by several data. Specific *p14*^*ARF*^ germline defects were previously reported in a family with melanoma and neural tumours and in a patient with multiple melanomas (Randerson-Moor *et al*, 2001; Rizos *et al*, 2001b). In addition, some of the *INK4a-ARF* germline mutations found in melanoma–prone families have been shown to affect p14^ARF^ function (Rizos *et al*, 2001a). Our mutation lies within the C-terminal p14^ARF^ nucleolar localisation domain, which is essential for full p14^ARF^ activity (Rizos *et al*, 2000). On the other hand, this mutation lies at a nonconserved codon between mice and human ARF protein, and therefore, may be a rare nonpathogenic variant.

The third variant is a *p16*^*INK4a*^ substitution located 25 bp upstream of the ATG, in the p16^INKa^ 5′ UTR and was detected in one of 14 patients with an NPIM. Germline mutations in critical regions of the p16^INKa^ promoter could reduce or abolish promoter function, resulting in a genetic predisposition to disease. Yet, to date, only three variants localised in the 5′UTR of p16^INKa^ (−14C>T, −33 G>C, −34 G>A) have been characterised in melanoma patients (Liu *et al*, 1999) (Hashemi *et al*, 2000) (Auroy *et al*, 2001), of which only the latter one has a proved functional effect (Liu *et al*, 1999). This particular mutation localised 34 bp upstream from the ATG translation initiation codon, creates an aberrant initiation codon, and has been detected in two MPM patients and in two melanoma families. In our case, it should be noted that the −25 C>T substitution was previously found in a skin squamous carcinoma from a xeroderma pigmentosum patient (Soufir *et al*, 2000), suggesting that it could be pathogenic. On the other hand, this mutation was not observed in a mutational screening of the p16^INKa^ promoter in 109 melanoma families (Harland *et al*, 2000), therefore raising the possibility of a rare polymorphism, and indicating the need for functional studies of p16^INKa^ expression in order to determine whether or not promoter single-nucleotide polymorphisms are pathogenic.

No germline mutation of *Cdk4* exon 2 was detected in any melanoma patient. This confirms previous studies performed in melanoma families (Goldstein *et al*, 2002), and further shows that the *Cdk4* gene is very rarely involved in genetic predisposition to melanoma.

We found no germline deletion of the *INK4a-ARF* locus in 71 melanoma patients, indicating that constitutional inactivation of this locus by deletion is not a frequent mechanism in genetic predisposition to melanoma.

In conclusion, our study confirms that germline mutations of the *INK4a-ARF* gene are predominantly involved in genetic predisposition to familial melanoma, particularly in large multicase melanoma families or in families comprising a member affected with multiple melanomas. Other conditions, despite suggesting a genetic predisposition to melanoma, rarely show *INK4a-ARF* germline mutations. Our findings are in accordance with the Melanoma Genetics Consortium, which considers that melanoma patients with or without stringent familial criteria should not be tested outside of defined research protocols (Kefford *et al*, 1999, 2002).

Besides these major melanoma-predisposing genes, other genetic predisposition factors exist. Firstly, polygenic inheritance in combination with environmental factors such as high sun exposure has been shown in several studies, depending upon polymorphisms located on genes controlling DNA repair (Winsey *et al*, 2000), pigmentation (Palmer *et al*, 2000), and reactive oxygen detoxification pathways (Strange *et al*, 1999; Kanetsky *et al*, 2001). Among these, loss of function variants of the human melanocortin-1 receptor gene, which plays a crucial role in pigmentation (Valverde *et al*, 1995; Bastiaens *et al*, 2001), seems to have an important role in determining melanoma risk (Palmer *et al*, 2000; Kennedy *et al*, 2001). Secondly, the possibility of mutations in additional, as yet unidentified highly penetrant melanoma-predisposing genes is still a research tool.
